# Cardiac papillary fibroelastomas: Unveiling a rare right atrial presentation with surgical insights—A case report and review of the literature

**DOI:** 10.1002/ccr3.9207

**Published:** 2024-08-06

**Authors:** Delaram Narimani Davani, Azin Alizadehasl, Azam Yalameh Aliabadi, Aida Bazrgar, Hamidreza Pouraliakbar, Seyedeh Fatemeh Hosseini Jebelli, Soroush Najdaghi, Yasamin Afsari Zonooz

**Affiliations:** ^1^ Heart Failure Research Center Cardiovascular Research Institute, Isfahan University of Medical Science Isfahan Iran; ^2^ Cardio‐Oncology Research Center, Rajaie Cardiovascular Medical and Research Center Iran University of Medical Sciences Tehran Iran; ^3^ Student Research Committee, School of Medicine Shiraz University of Medical Sciences Shiraz Iran; ^4^ Department of Radiology, Rajaie Cardiovascular Medical and Research Centre Iran University of Medical Sciences Tehran Iran

**Keywords:** cardiac papillary fibroelastoma, right atrium, transthoracic echocardiography

## Abstract

Cardiac papillary fibroelastomas (CPF) are rare, benign tumors with thromboembolic potential. We present a 40‐year‐old male with a right atrial CPF, referred with acute chest pain. Advanced imaging and surgical excision with tricuspid valve repair were crucial, emphasizing the need for early detection and intervention in symptomatic and asymptomatic cases.

## 1 BACKGROUND

Cardiac tumors are relatively uncommon compared to other forms of cardiovascular disease and malignancies affecting different body organs.^1^ Among primary cardiac tumors, cardiac papillary fibroelastomas (CPF) account for approximately three‐quarters of all cardiac valvular malignancies, making them the second most common type of primary cardiac tumor.^2^ The prevalence of these benign endocardial papillomas is estimated to range from 0.0017% to 0.33% based on the autopsy series.^3^ While only 2% of all CPFs originate in the right atrium (RA), the left heart accounts for more than 95% of CPF cases.^4^ Fibroelastomas present a unique echocardiographic profile, featuring a stalk connected to a cardiac valve with a centralized cord and radiating fronds. Transesophageal echocardiogram (TEE) is the recommended imaging modality, with surgical resection as the preferred treatment.^5^ Despite their rare incidence and the histologically benign nature of CPF tumors, they may cause potentially fatal complications such as acute valvular dysfunction, thromboembolism, stroke, and unexpected sudden death.^2^ Here, we discuss a unique case of CPF tumor arising from the RA, presenting with pleuritic chest pain, its challenging management, and treatment strategies, as well.

## 2 CASE PRESENTATION

A 40‐year‐old male, without remarkable medical history, presented to our hospital on December 2023, following an evaluation for acute‐onset right‐sided pleuritic chest pain. The pain is exacerbated by deep breathing and movement and the patient describes it as sharp and stabbing discomfort. The patient is evaluated for vital signs, and a focused medical history is obtained. Physical examination reveals localized tenderness upon palpation of the right chest wall during inspiration; no cardiovascular risk factor was obtained based on the patient's claim.

### 2.1 Investigations

**FIGURE 1:**
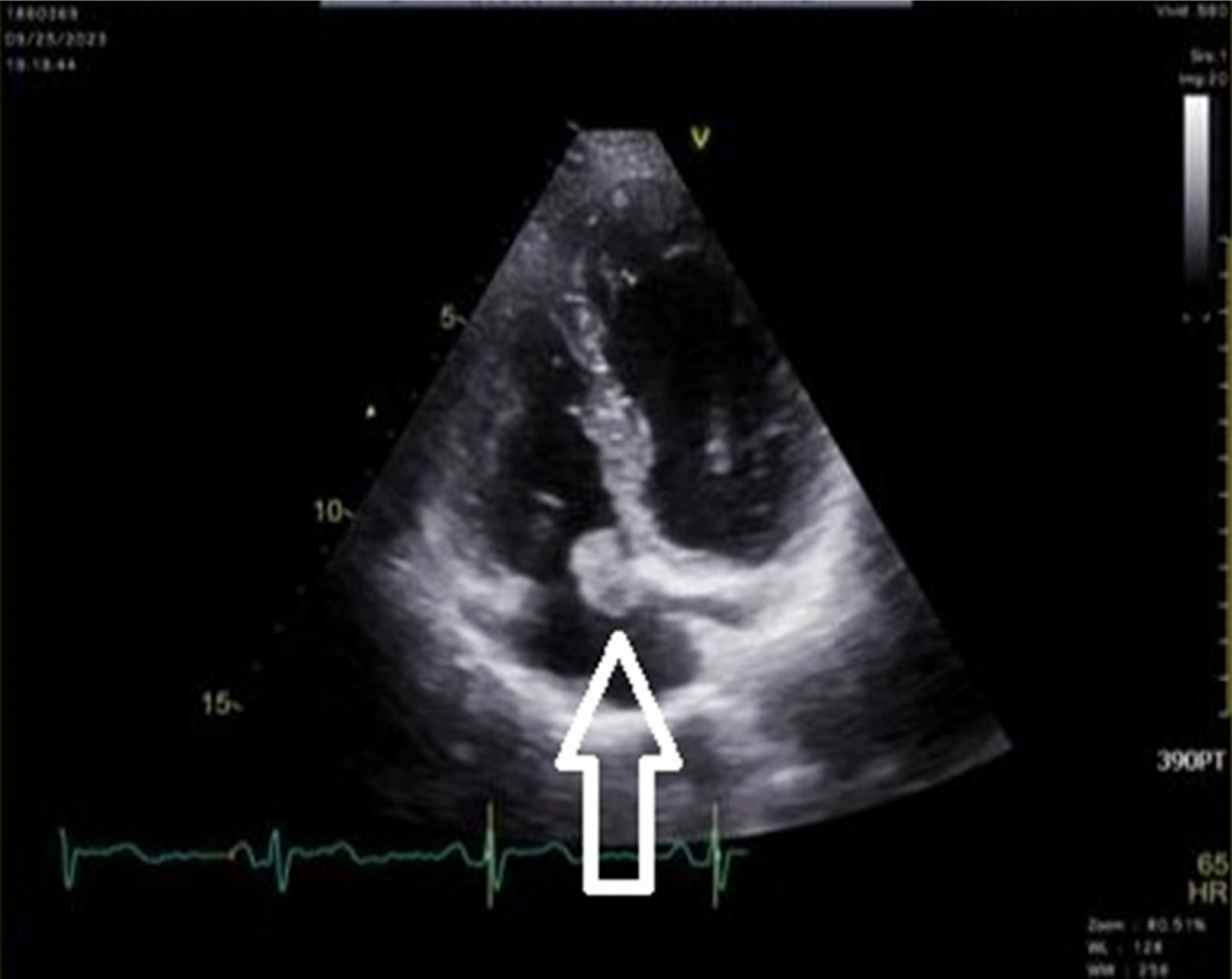
TTE of a 44‐year‐old male with pleuritic chest pain before surgery (4‐chambers view). Large oval‐shaped mobile, gelatinous, and homogenous echodensity (2.0 cm× 1.6 cm) is seen in RA attached to the posterior part of TV annulus just adjacent to CS orifice, attachment to posterior leaflet could not be evaluated in TTE, mostly in favor of myxoma (white arrow). Notably, there are no significant valvular abnormalities or indications of pericardial effusion. Mild AI, MR, PI, and mild to moderate TR are seen, along with LVEF = 55%. AI, aortic insufficiency; CS, coronary sinus; LVEF, left ventricular ejection fraction; MR, mitral regurgitation; PI, pulmonary insufficiency; RA, right atrium; TR, tricuspid regurgitation; TTE, transthoracic echocardiography; TV, tricuspid valve.

**FIGURE 2:**
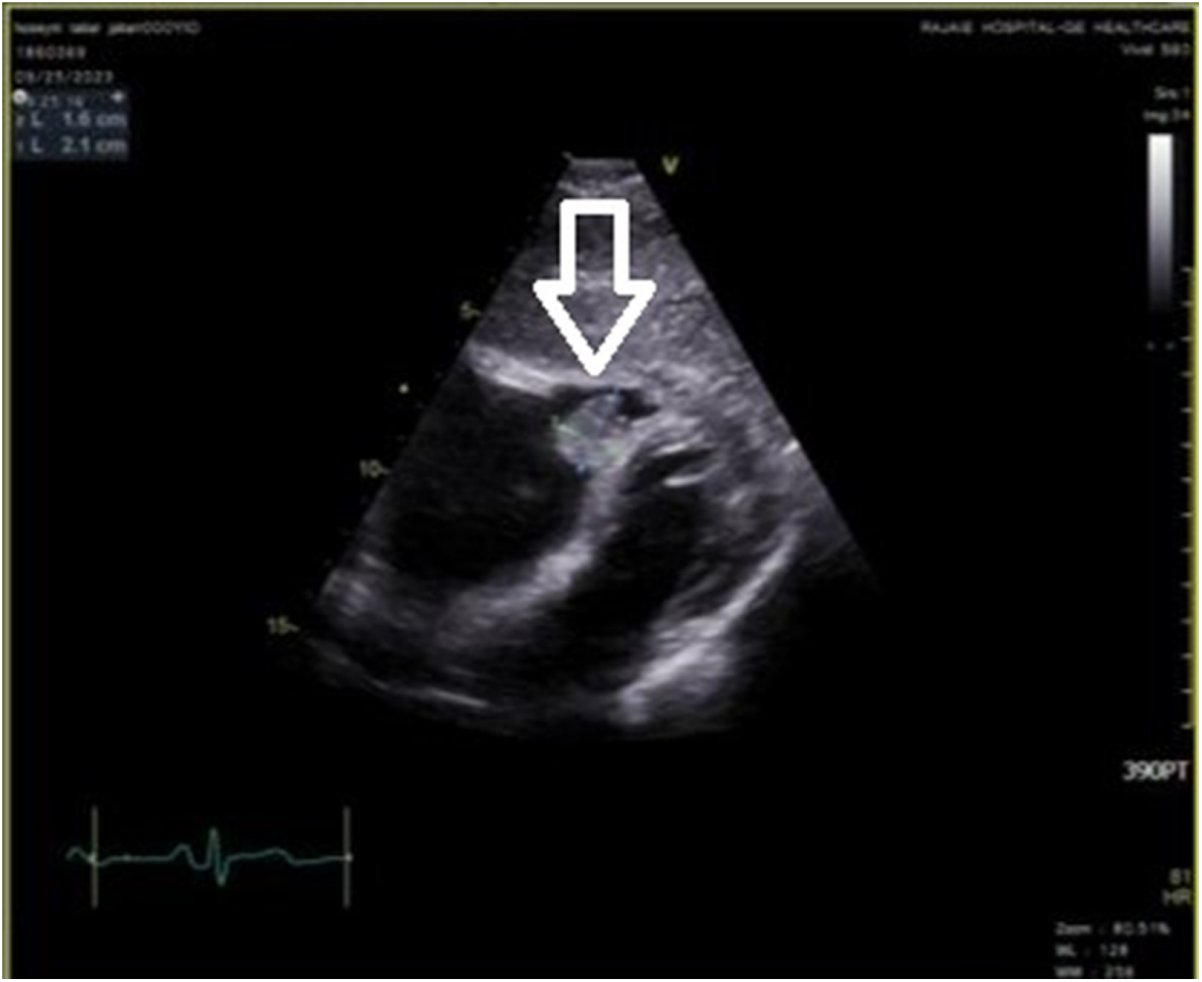
TTE of a 44‐year‐old male with pleuritic chest pain, before surgery (subcostal view). The mass is visualized in the parasternal short axis view, with the cystic component being more prominently featured (white arrow). Importantly, no evidence indicates attachment of the mass to the tricuspid valve or the right ventricular outflow tract. TTE: transthoracic echocardiography.

The electrocardiogram (ECG) and systolic function, following transthoracic echocardiography (TTE), revealed normal sinus rhythm and left ventricular ejection fraction (LVEF) of 55% (Figures 1, 2). Subsequent evaluation via transesophageal echocardiography (TEE) confirmed the presence of a sizable, oval‐shaped, mobile, and heterogeneous mass in the RA, measuring 2.0 × 1.6 cm. The mass was affixed to the RA wall with a small stalk, situated just above the posterior tricuspid valve (TV) annulus, occasionally protruding toward the TV orifice. No masses were observed in other cardiac chambers, and no abnormalities in wall motions or other structural aspects were reported.

**FIGURE 3:**
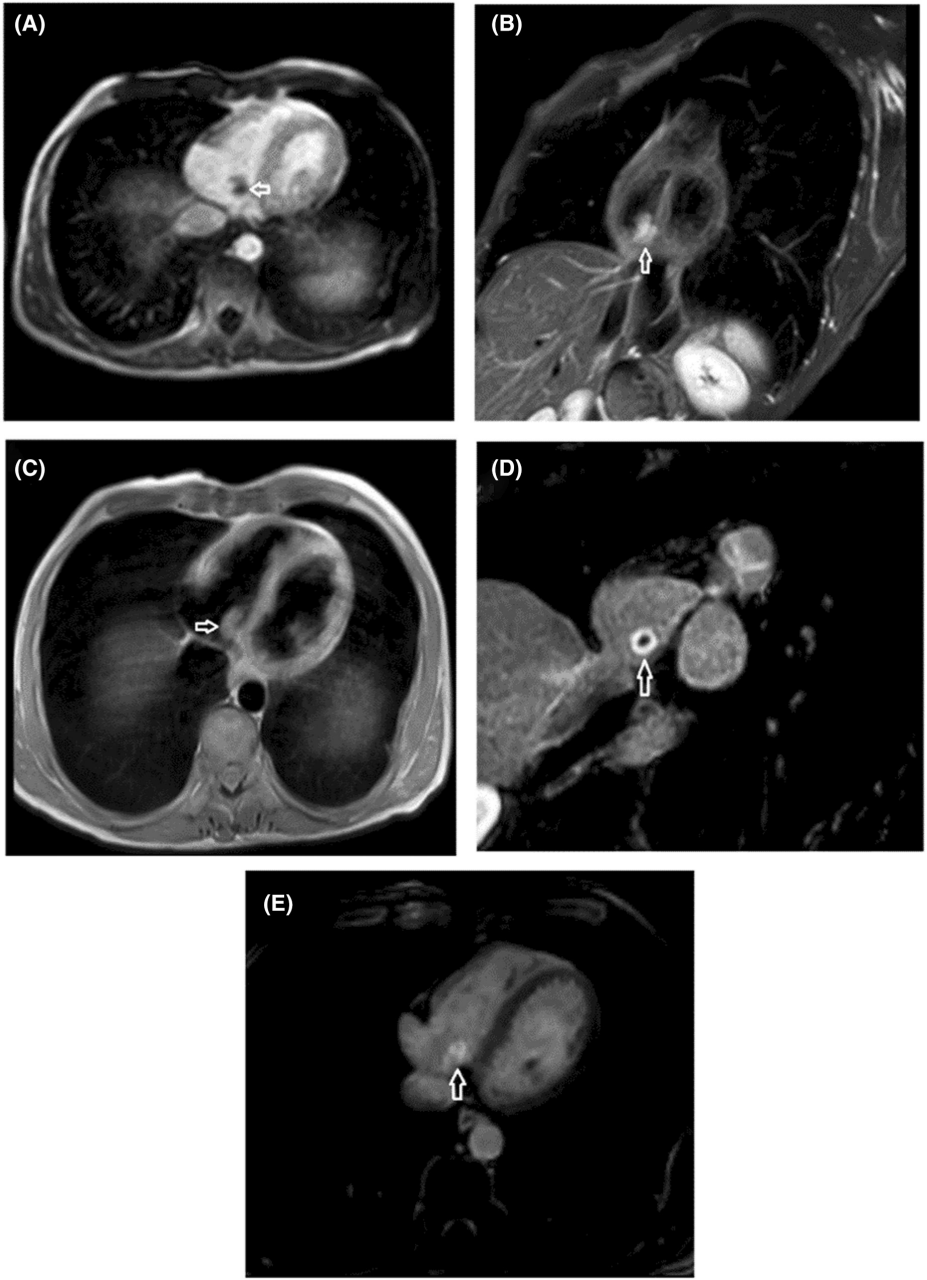
CMR imaging of a well‐defined, lobulated tumor (arrow) at the atrial side of the tricuspid valve's posterior leaflet. (A) Hyperintense tumor in short‐axis view SSFP Cine. (B) Hyperintense tumor in short‐axis view STIR. (C) Isointense tumor in axial T1W. (D, E) Intense peripheral enhancement with central sparing (Thick Ring Enhancement) in axial and short‐axis LGE views. CMR, cardiac magnetic resonance; LGE, late gadolinium enhancement; SSFP, steady‐state free precession; STIR, short tau inversion recovery; T1W, T1‐weighted.

Supplementary cardiac magnetic resonance (CMR) imaging, specifically tailored for RA tumor assessment, resulted in LVEF = 59%, mild AI, and PI. It also represents There is a small 10 × 9 mm mobile mass attached to the RA wall and base of the TV, which is isointense in T1, hyper‐intense in T2 STIR sequences, shows normal perfusion, but heterogeneous peripheral enhancement in late gadolinium enhancement (LGE) sequence. These findings are suggestive of myxoma or fibroelastoma (Figure 3A–E).

Concurrently, routine clinical evaluations, including physical exams, labs, and abdominal sonography, consistently reported normal outcomes. This combined approach reinforces the localized nature of the cardiac papillary fibroelastoma, informing surgical planning.

### 2.2 Treatment

**FIGURE 4:**
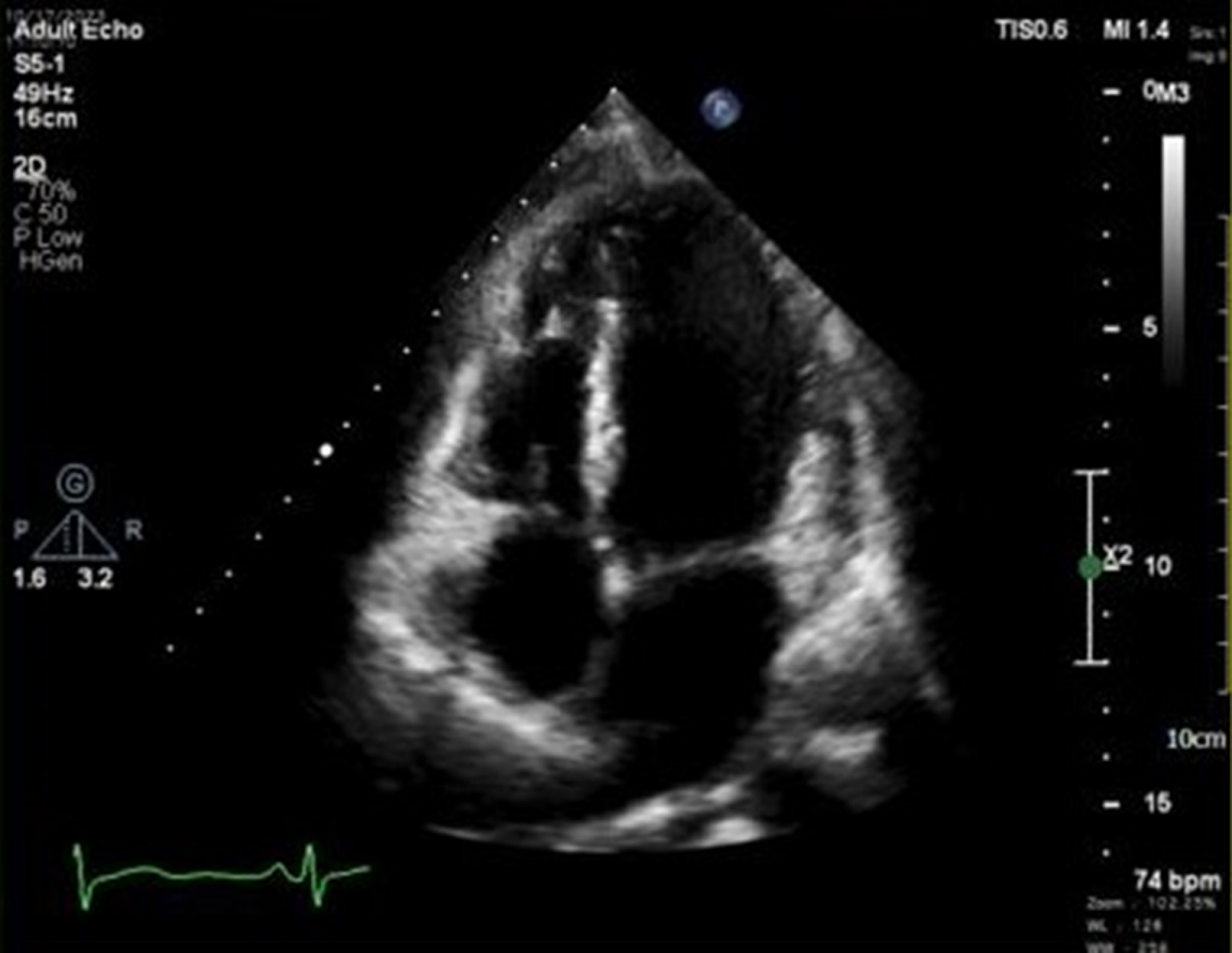
TTE of a patient with papillary fibroelastoma of RA, underwent mass resection and TV repair. Complete surgical excision resulted in the absence of residual mass or thrombus in the RA, with a normal size, and mild to moderate systolic dysfunction. Mild AI, MR, and TR are seen. Mildly thickened and dome‐shaped PV is reported. Also, mild pericardial effusion and hematoma without hemodynamic effect, besides the Left side pleural effusion are seen. AI, aortic insufficiency; MR, mitral regurgitation; RA, right atrium; TR, tricuspid regurgitation; TTE, transthoracic echocardiography; TV, tricuspid valve.

The patient underwent surgical intervention for the RA mass and TV repair through midline sternotomy in January 2024. Following meticulous prepping in sterile conditions and under general anesthesia, a midline incision was made, and a sternotomy was performed. Cannulation of the aorta, superior vena cava (SVC), and inferior vena cava (IVC) ensued. With the initiation of the pump and cardiac arrest, the RA was opened, revealing a 2 × 2 cm tumor on the posterior tricuspid valve, addressed through the bicuspidization method. Subsequent RA repair was conducted, and gradual weaning off the pump was achieved. A post‐repair TEE displayed no tricuspid regurgitation (TR) or residual issues, confirming successful intervention. Hemostasis was maintained, chest tubes were placed, and sternum closure ensued. Post‐surgery TTE indicated no residual RA mass but noted mild pericardial effusion and hematoma without hemodynamic impact (Figure 4).

### 2.3 Outcome and pathological evaluation

#### 2.3.1 Gross findings

The examination of the specimen labeled as “Right Atrial Mass” revealed five pieces of cream tissue, each exhibiting multiple small fronds measuring between 0.5 and 1.5 cm in diameter.

#### 2.3.2 Microscopic analysis

Microscopic examination disclosed multiple fronds of paucicellular avascular fibroelastic tissue, each lined by a single endocardium layer. Additionally, foci of hydropic changes were identified within the tissue.

#### 2.3.3 Final pathological diagnosis

The comprehensive examination aligns with a final diagnosis consistent with papillary fibroelastoma. This conclusion is further supported by identifying only a few villi exhibiting cores of concentric small, thin, and discontinuous elastic lamellae.

### 2.4 Follow‐up

The patient's postoperative course has been uneventful, marked by a smooth recovery without complications. To date, 3 months postoperatively, the patient continues to fare well without any complications or adverse events, affirming the success of the surgical intervention in ensuring a positive clinical outcome.

## 3 DISCUSSION

**FIGURE 5:**
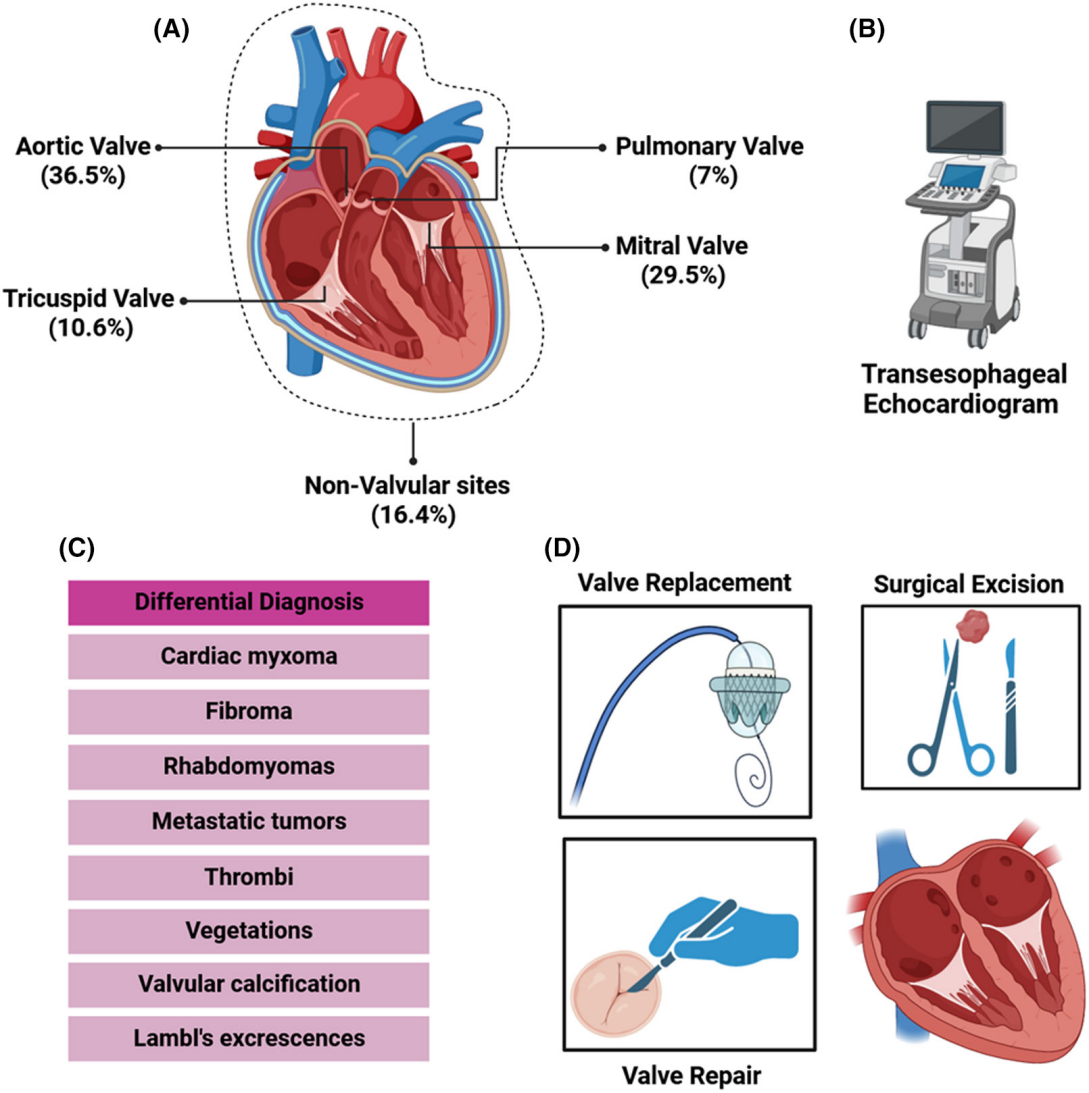
Summary diagram of the sites of involvement, diagnosis, and treatment of cardiac fibroelastoma. (A) Frequency of the fibroelastoma in various cardiac segments—valvular and non‐valvular. (B) The preferred diagnostic method in patients with fibroelastoma. (C) Differential diagnoses of cardiac fibroelastoma. (D) Available treatment strategy methods for cardiac fibroelastoma.

Primary cardiac tumors are infrequent, comprising about 0.019%–0.021% of cases, with CPF ranking as the second most prevalent (4.4%–8%) after cardiac myxomas. CPF predominantly affects males (55%), commonly emerging in individuals in their seventh decade.^6^ Figure 5A delves into the examination of the frequency distribution of the occurrence of CPF in various segments of the heart, based on a comprehensive analysis of reported data from 611 patients.^2^

### 3.1 Etiology and clinical manifestations

The etiology lacks clear risk factors, with proposed theories encompassing a microthrombus hypothesis, viral, traumatic, or congenital origins.^3^ Typical presentations involve neurologic deficits from stroke or transient ischemic attacks, while asymptomatic cases are often incidentally discovered during unrelated surgical interventions.^3^ CPFs predominantly affect the left ventricle, with right‐sided occurrences being relatively rare.^3^ In most cases, right‐sided CPFs do not cause any symptoms until they get enlarged and block the blood flow.^1^ Presenting our case, the instance of the RA involvement with TV, and the manifestation of pleuritic pain, highlights a rare site and symptom presentation for the tumor.

### 3.2 Differential diagnosis

The differential diagnosis of CPF highlights distinguishing features from other cardiac conditions. It covers comparisons with cardiac myxoma, fibroma, rhabdomyoma, metastatic tumors, thrombi, vegetations, valvular calcification, and Lambl's excrescences^2^ (Figure 5C).

From histological structure, CPF is characterized by a distinctive frond‐like, or sea‐anemone‐like, structure. It consists of a central core of dense collagen and elastic fibers, covered by a single layer of endothelial cells. This frond‐like architecture is unique and differentiates CPF from other cardiac tumors such as myxomas, which have a more gelatinous, myxoid stroma, and rhabdomyomas, which consist of striated muscle cells.^7^ Moreover, CPF primarily occurs on the endocardial surfaces of the heart valves, most commonly the aortic and mitral valves. In contrast, other cardiac tumors like myxomas are often found in the atrial chambers of the heart, particularly the left atrium, attached to the interatrial septum.^8^

### 3.3 Imaging diagnosis

CPF cases are often incidentally discovered during unrelated evaluations. ECG may show nonspecific findings or atrial arrhythmias.^2^ Initial assessment involves TTE, while comprehensive evaluation necessitates TEE (Figure 5B). Imaging typically reveals small, mobile valvular masses with fluttering or prolapsing motions.^2^ Chest roentgenograms may indicate cardiac chamber enlargement, and pulmonary signs if mitral valve obstruction occurs.^2^ Calcification, rarely visible on routine radiography, may appear in computed tomography (CT), but echocardiography is preferred for real‐time imaging of small structures.^2^ CMR provides multiple plane views and soft‐tissue details.^2^ Cardiac catheterization is rarely needed, reserved for cases where noninvasive methods are inconclusive or when another cardiac condition is suspected.^2^ It may reveal intracavity tumors, and coronary abnormalities, and pose added risks due to potential embolism.^2^ We have utilized three modalities (TTE, TEE, and CMR) for both initial diagnosis and differentiation from another possible diagnosis, in our case.

### 3.4 Treatment

The therapeutic landscape for CPF involves a tailored approach, with recommendations for surgical resection extending to 64.1% of cases, especially for tumors exceeding 1 cm in size and exhibiting notable mobility.^2^, ^9^ However, Guglielmo et al.^10^ recommended surgical excision of mobile lesions, despite their size, due to the increased risk of thromboembolic complications.

Recurrence, a rare phenomenon, documented at 0.04% post‐surgical resection, underscores the efficacy of this intervention.^5^, ^11^ Moreover, patients undergoing surgical removal experience a commendable 30% increase in overall survival rates during the initial 7 years following the procedure compared to non‐operative management.^11^

Navigating asymptomatic cases introduces nuanced considerations, with tumor mobility serving as a crucial determinant.^12^ Surgical excision is recommended for mobile lesions, while conservative strategies, such as anticoagulation and periodic echocardiographic observation, are suitable for non‐mobile tumors.^12^ This approach aligns to minimize embolic risks and optimize patient outcomes. However, it is recommended to monitor right‐sided lesions and perform surgical removal only if they manifest symptoms.^13^ Yet, making decisions about which half of the heart lesions, needed surgical removal—by considering echocardiographic evidence (in terms of size and/or mobility) and the patient's thromboembolic risk—requires a thorough investigation (Figure 5D). In our case, the CPF was resected via sternotomy, and the TV was repaired by the Bicuspidization method.

Additionally, antiplatelet agents, such as aspirin, are recommended for patients with CPF primarily to reduce the risk of thromboembolic events. CPFs, particularly those located on heart valves, have a high potential for causing embolic events due to their friable and mobile structure.^8^ These agents help mitigate this risk by inhibiting platelet aggregation, which can contribute to the formation of thrombi that may embolize. While antiplatelet therapy is common, anticoagulant therapy may also be considered, especially if there is evidence of thrombus formation or a high risk of embolic events. Some authors have noted the use of anticoagulants in cases where surgery is not an option or in right‐sided heart tumors with potential pulmonary embolism risk.^14^ So, the choice between antiplatelet and anticoagulant therapy can depend on the individual patient's risk factors and overall health condition.^2^

### 3.5 Literature review

A comprehensive search was conducted using PubMed and Google Scholar databases to identify relevant case reports focusing on the nonvalvular right atrial CPF. The search strategy utilized the following keywords: “Cardiac Papillary Fibroelastoma” AND “Right Atrium.” All English‐language case reports published up to March 2024 were included in the search.

A total of 23 articles (with 26 cases) were reported (Table 1). Most cases (57.7%) were male and the mean age of afflicted patients was 69.15 ± 14.43 (mean ± S.D.). The lesions were different in size (ranging from 4 mm^15^ to 46 mm^16^), and the site of attachment (from “Inferior ridge of the right atrial appendage” to “Chiary network”). Surgical removal was the primary choice for nearly 77% of cases; however, in some cases (11.5%), due to the size of the mass, patient hemodynamic conditions, and other factors, periodic follow‐up may be preferred^16^, ^35^ [reference 13, cases 2 and 3]. However, when surgery is not feasible or refused, long‐term antiplatelet therapy is recommended, due to the risk of superimposed thrombi.^11^

**TABLE 1:** Reported cases of nonvalvular right atrial papillary fibroelastoma.

Author (year)	Gender, age	Presentation	Size (mm)	Site of attachment	Treatment decision
Schiller et al. (1970)^15^	Female, 92	Incidental	10	Anterior right atrial endocardium	Patient died
	Male, 72	Incidental	4	Anterior right atrial endocardium	Patient died
Schwinger et al. (1989)^16^	Female, 83		46	Right atria1 appendage	Digoxin (for ventricular rate control)
Wasdahl et al. (1992)^17^	Male, 85	Autopsy	8	Chiari network	Patient died
Gallas et al. (1993)^18^	Male, 69	Incidental	25 × 20	Lateral atrial wall	Surgical removal
Crestanello et al. (2002)^19^	Female, 86	Syncope	40	Interatrial septum	Surgical removal
Singh et al. (2004)^20^	Male, 75	Asymptomatic	25 × 25	Right atrial free wall	Surgical removal
Hindupur et al. (2005)^21^	Male, 77	Syncope	26 × 23	Right atrial free wall	Surgical removal
Nagano et al. (2005)^22^	Female, 68	Incidental	20	Interatrial septum	Surgical removal
Gabbieri et al. (2006)^23^	Female, 70	Recurrent pulmonary embolism	10 × 10	Right atrial free wall	Surgical removal
Lotto et al. (2006)^24^	Female, 60	Coronary artery disease	20 × 22	Right atrial free wall	Surgical removal
Lauten et al. (2007)^25^	Female, 66	Asymptomatic	28 × 25	Right atrial free wall	Surgical removal
Kim et al. (2007)^26^	Female, 62	Incidental	N/A	Lateral atrial wall	Surgical removal
Latif et al. (2008)^27^	Male, 41	Infective endocarditis	12 × 20	Chiari network	Surgical removal
Abad et al. (2008)^28^	Male, 60	Transient ischemic attack	20 × 15	Atrial muscular endocardium	Surgical removal
Maybury et al. (2009)^29^	Male, 74	Presyncope	10 × 10	Edge of the right atrial wall	Surgical removal
Nwiloh et al. (2011)^30^	Male, 73	Transient ischemic attack	33 × 30	Interatrial septum	Surgical removal
Marboeuf et al. (2012)^31^	Male, 65	Pulmonary embolism	30 × 28	Right atrial free wall	Surgical removal
Emad et al. (2013)^32^	Male, 61	Incidental	20 × 22	Interatrial septum	Surgical removal
Ijaz Haider et al. (2015)^33^	Male, 81	Incidental	11–12	Junction of right atrial wall and superior vena cava	Surgical removal
Arata Muraoka et al (2015)^34^	Female, 70	Exertional dyspnea	Not available	Right atrial free wall	Surgical removal
Giuseppe Pilato et al. (2020)^35^	Male, 75	Incidental	10	Inferior ridge of the right atrial appendage	Surgical removal
	Female, 79	Incidental	5	Border of the right atrial appendage	Dual antiplatelet therapy (aspirin 300 mg and clopidogrel 75 mg) and statins
	Female, 79	Incidental	12	On the ridge of the right atrial appendage	1‐year‐follow up with Apixaban 5 mg twice/daily
Nana Hu et al. (2021)^12^	Male, 54	Incidental	15.6 × 12.4	Chiari network	Surgical removal
Le Ho et al. (2021)^36^	Male, 21	Pulmonary embolism	34 × 34 × 20	Interatrial septum	Surgical removal

## 4 CONCLUSION

In conclusion, our report illustrates the rare case of papillary fibroelastoma in RA, with nonspecific presentation. While CPFs are infrequent and histologically benign, they can result in significant clinical complications, particularly thromboembolic events. Echocardiography, especially TEE, combined with CMR imaging, offers the level of structural detail required to determine the precise location and extent of both anatomical and hemodynamic involvement. Surgical removal of the fibroelastoma is recommended for survival advantage, even in asymptomatic patients. Those who are not eligible for surgical intervention should receive long‐term anticoagulation therapy, despite the absence of guidelines endorsing this approach.

## AUTHOR CONTRIBUTIONS

**Delaram Narimani Davani:** Writing – original draft; writing – review and editing. **Azin Alizadehasl:** Project administration; supervision. **Azam Yalameh Aliabadi:** Resources; validation. **Aida Bazrgar:** Investigation. **Hamidreza Pouraliakbar:** Resources; visualization. **Seyedeh Fatemeh Hosseini Jebelli:** Writing – review and editing. **Soroush Najdaghi:** Writing – review and editing. **Yasamin Afsari Zonooz:** Writing – review and editing.

## FUNDING INFORMATION

None.

## CONFLICT OF INTEREST STATEMENT

The authors declare no conflicts of interest.

## CONSENT

Written informed consent was obtained from the patient to publish this report in accordance with the journal's patient consent policy.

## Data Availability

All data generated or analyzed during this study are included in this published article.
